# Design of skin islands for a myocutaneous serratus anterior free flap—An anatomical study and clinical implication for pharyngeal reconstruction after laryngopharyngectomy

**DOI:** 10.1111/coa.13257

**Published:** 2018-12-11

**Authors:** Stefan J. Janik, István Paraszti, Lena Hirtler, Rudolf Seemann, Hannes Traxler, Wolfgang Weninger, Boban M. Erovic

**Affiliations:** ^1^ Department of Otorhinolaryngology, Head and Neck Surgery Medical University of Vienna Vienna Austria; ^2^ Department of Otorhinolaryngology University Hospital St. Pölten St. Pölten Austria; ^3^ Center of Anatomy and Cell Biology Medical University of Vienna Vienna Austria; ^4^ Department of Cranio‐Maxillofacial and Oral Surgery Medical University of Vienna Vienna Austria; ^5^ Institute for Head and Neck Diseases Evangelical Hospital Vienna Vienna Austria

**Keywords:** flap design, laryngopharyngectomy, myocutaneous free flap, pharyngeal reconstruction, serratus anterior free flap

## Abstract

**Objectives:**

The main purpose of this study was to evaluate flap size and flap design of skin islands in myocutaneous serratus anterior free flaps (SAFFs) in fresh cadavers and to further investigate whether myocutaneous SAFFs are suitable flaps for pharyngeal reconstruction after laryngopharyngectomy.

**Methods:**

Dissection and injection of methylene blue were performed in 20 hemithoraces of 13 fresh cadavers to evaluate flap size and location of skin islands. Based on these pre‐clinical data, we performed pharyngeal reconstruction with myocutaneous SAFF in five patients after laryngopharyngectomy.

**Results:**

Perfused skin paddles were found in all specimens with a mean size of perfused skin islands of 85.6 ± 49.8 cm^2^. Lengths and widths of skin islands ranged from 10‐21 cm and 6‐20.5 cm respectively. Flap size did not significantly differ between males and females (*P* = 0.998), left compared to right hemithoraces (*P* = 0.468) and between paired specimens (*P* = 0.915). All skin islands were found within the upper 29.3%‐51.7% of hemithorax (calculated from axilla to costal arch), and between latissimus dorsi muscle posteriorly and anterior axillary line anteriorly. Accordingly, myocutaneous SAFFs were used for pharyngeal reconstruction after laryngopharyngectomy in five patients with advanced hypopharyngeal carcinomas. Three patients had uneventful courses, while one patient developed immediate intraoperative flap loss and another patient developed partial necrosis of SAFF on postoperative day 7.

**Conclusion:**

Skin islands of SAFF have reliable blood supply, which allow harvest of large myocutaneous SAFFs that can be used also for pharyngeal reconstruction after laryngopharyngectomy.

**Keypoints**

- Serratus anterior free flap.
- Myocutaneous free flap.
- Flap design.
- Laryngopharyngectomy.
- Pharyngeal reconstruction.

## 1 INTRODUCTION

The serratus anterior free flap (SAFF) was first described in 1982 by Takayanagi and Tsukie for reconstruction of lower limb defects.^1^ In the following, the SAFF has found many applications in head and neck reconstruction, including reconstruction of the oral cavity, the scalp, the skull base, craniofacial defects or the oesophagus.^2^, ^3^, ^4^, ^5^, ^6^, ^7^

The serratus anterior muscle originates anteriorly on the first nine ribs and inserts at the medial border of the scapula.^6^ It derives its main vascular supply from the thoracodorsal artery (TDA), representing a branch of the subscapular artery (SA), which in turn originates from the axillary artery (AA).^6^ However, numerous anatomical variations are possible that have been already described in literature. Accordingly, vascular supply can directly arise from the AA,^8^ the intercostal artery^9^, ^10^ or the lateral thoracic artery.^2^, ^11^

Particularly for reconstruction of the head and neck, the SAFF offers some significant advantages, including a long and reliable vascular pedicle, the thinness and pliability of the flap, ease of harvest and a low donor site morbidity. Moreover, due to its central localisation, the SAFF has a low risk for peripheral arterial vascular disease.^6^ Although the exact extent and localisation of perfused skin paddles for myocutaneous SAFF have not been defined so far, the SAFF has been successfully used as a myocutaneous free flap for the reconstruction of the face,^2^ the head and neck^12^ or the lower limb.^13^

Hence the main purpose of the study was to perform an anatomical study in order to evaluate the optimal area and size for the harvest of skin islands perfused by the thoracodorsal artery and to define applicable recommendations for clinical routine. Based on these pre‐clinical data, we additionally report on our first clinical experiences of pharyngeal reconstruction using a myocutaneous SAFF after laryngopharyngectomy.

## 2 MATERIAL AND METHODS

The anatomical part of the study was performed at the Center of Anatomy and Cell Biology of the Medical University of Vienna. On the other hand, the clinical part of the study was done at the Department of Otorhinolaryngology, Head and Neck Surgery of the Medical University of Vienna.

### 2.1 Anatomical study

**Figure 1:**
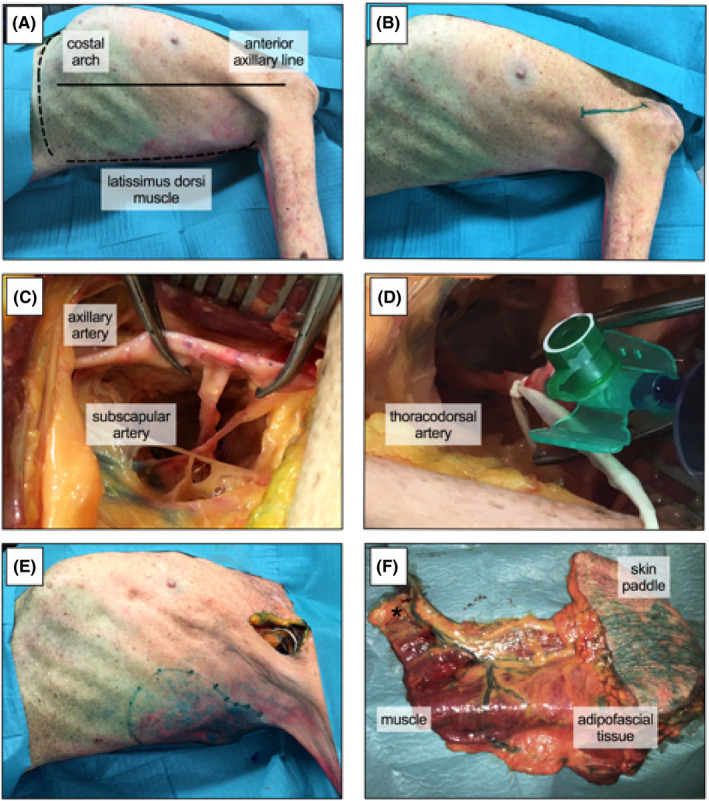
Dissection of myocutaneous serratus anterior free flap. Dissection was performed in supine position with 90° abduction. Anterior axillary line, costal arch and latissimus dorsi muscle were indicated as landmarks (A). A vertical skin incision (~10 cm) was done within the anterior axillary line (B). Fascia and pectoralis major muscle were further separated (not shown) and axillary artery was identified. Next subscapular artery (C), which ends up in thoracodorsal artery (D), was cannulated. After injection of 40‐60 mL methylene blue, stained skin islands were documented and analysed (E). Finally a myocutaneous serratus anterior free flap was harvested (F). *Asterisk indicates vascular pedicle

Twenty hemithoraces, including the upper limb, were dissected from 13 fresh cadavers (five female, eight male). Six specimens were excluded due to former operations on the lateral thoracic wall or chest (eg, insertion of pacemaker or thoracotomy). The specimens were obtained from voluntary donors who consented during lifetime to donate their body for research and teaching purpose to the Center for Anatomy and Cell Biology at the Medical University of Vienna. Specimens were dissected in supine position with full abduction (90°) of the upper limb. First, a vertical skin incision (~10 cm) within the anterior axillary was done. Second, the pectoralis major muscle was separated and the AA was identified at the level of its transition into the brachial artery. Next, the SA was found in the third part of the AA, which directly ends up in the TDA. After the TDA was cannulated, 40‐60 mL methylene blue was injected (Figure 1). Finally, size and localisation of skin islands were evaluated.

#### 2.1.1 Assessment of skin islands

Two different methods were used for assessing and analysing skin islands. First, a coordinate system was used consisting of anatomical reference lines (posterior axillary line, anterior axillary line, midclavicular line) and landmarks (costal arch, axilla, latissimus dorsi muscle, nipple) to describe and analyse localisation of skin islands. The distance between axilla and costal arch (ACA), measured within anterior axillary line, has been used as surrogate marker for length of hemithorax. Moreover, distances between costal arch to the inferior border (CAIB) and axilla to the superior border (ASB) of stained skin islands were measured to assess the vertical position of skin flap. Additionally, the horizontal extent of stained skin island was evaluated with regard to latissimus dorsi muscle and posterior axillary line posteriorly and nipple with midclavicular line anteriorly. We used a 1.0 mm ruler to measure the longest vertical (length) and horizontal (width) diameter of stained skin islands. After all variables were measured, stained skin islands were marked and photographed. In a second step, all photos were processed with imagej software (open source Java image processing program) and size of marked and stained skin islands were finally assessed.

### 2.2 Clinical implications

Based on our pre‐clinical anatomical results, we used myocutaneous SAFF for pharyngeal reconstruction in five patients who underwent laryngopharyngectomy due to a stage IV hypopharyngeal squamous cell carcinoma (SCC). All patients were treated at the Department of Otorhinolaryngology, Head and Neck Surgery, Medical University of Vienna, between February 2015 and March 2017. Flap harvest and reconstruction were done by the same head and neck surgeon (BE). Mean and median follow‐up were 13.8 and 10.4 months respectively. We used flap failure, occurrence of pharyngocutaneous salivary fistula (PCF), distal stenosis and swallowing function as main outcome parameters.

### 2.3 Statistical methods

spss software (version 21; IBM SPSS Inc, Chicago, IL, USA) was used for statistical analysis of data. All data are indicated as mean ± standard deviation (SD) within result section. Unpaired students *t* test was used to compare means of normally distributed variables, while Chi‐square test was performed to investigate the association between nominal variables. All tests were two‐sided and *P*‐values below 0.05 were considered as statistically significant.

### 2.4 Ethical considerations

Ethical approval was obtained from the ethics committee of the Medical University of Vienna prior to enrolment. Fresh cadavers for the anatomical part of the study were obtained from voluntary donors who consented during lifetime to donate their body for research and teaching purpose to the Center for Anatomy and Cell Biology at the Medical University of Vienna.

## 3 RESULTS

### 3.1 Anatomical study

#### 3.1.1 Flap size

**Figure 2:**
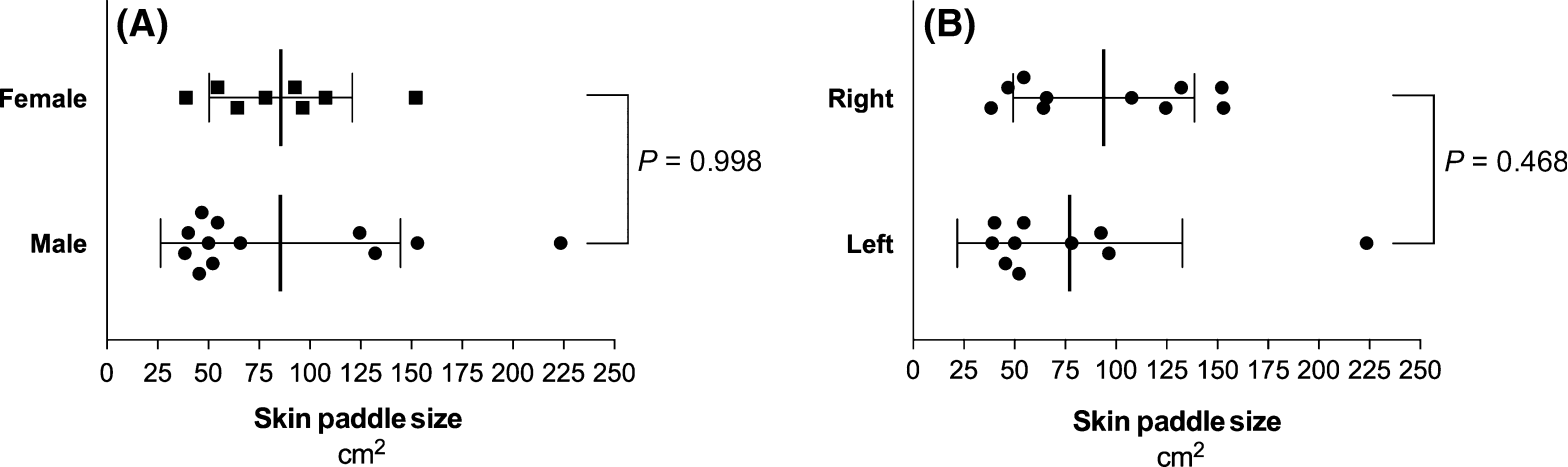
Size of skin islands according to sex and side. Size of skin islands was not significantly different in female compared to male specimens (A) or in left compared to right hemithoraces (B). Unpaired students *t* test was performed and mean ± standard deviation is indicated

We dissected and analysed 10 left and 10 right specimens of seven paired and six unpaired hemithoraces. Perfused skin islands were found in all specimens (20 out of 20). Mean length and width of skin islands were 15.5 ± 9.9 cm (range 10‐21 cm) and 10.9 ± 13.9 cm (range 6‐20.5 cm). Mean area of stained skin islands was 85.6 ± 49.8 cm^2^ with a range of 38.4‐223.6 cm^2^ (Table 1). Despite the great diversity of stained skin islands, there was no significant difference between female compared to male cadavers (85.6 ± 35.3 cm^2^ vs 85.5 ± 59.1 cm^2^; *P* = 0.998) or left compared to right hemithoraces (93.9 ± 44.7 cm^2^ vs 77.2 ± 55.5 cm^2^; *P* = 0.468; Figure 2). Additionally, area of stained skin islands did not significantly differ in left compared to right paired specimens (81.6 ± 65.8 cm^2^ vs 84.0 ± 43.9 cm^2^; *P* = 0.915).

**Table 1:** Skin islands of myocutaneous serratus anterior free flap

Case	Sex	Paired	Side	Flap size		
				Length cm	Width cm	Area cm^2^
1	M	Yes	R	15	9	124.5
2	M	Yes	L	18	20.5	223.6
3	M	Yes	L	15	12.5	45.5
4	M	Yes	R	10	9.5	46.8
5	M	No	L	11	6	50.1
6	F	No	L	15	13	96.5
7	F	No	L	15	8	54.5
8	M	No	R	15	11	132.2
9	F	Yes	R	14.5	14	107.7
10	F	Yes	L	20	16	39.0
11	M	No	R	21	17	65.8
12	M	No	R	12.5	8	153.0
13	F	Yes	R	15.5	11	64.3
14	F	Yes	L	21	11	92.6
15	M	Yes	L	20	8	52.2
16	M	Yes	R	12	6.5	54.5
17	F	Yes	R	16	10	152.2
18	F	Yes	L	16	10	78.1
19	M	Yes	R	13.5	8	38.4
20	M	Yes	L	14	8	40.1

F, Female; L, Left; M, Male; R, Right.

The maximum length (cm), width (cm) and area (cm^2^) of stained skin islands are indicated.

#### 3.1.2 Localisation of flap

**Figure 3:**
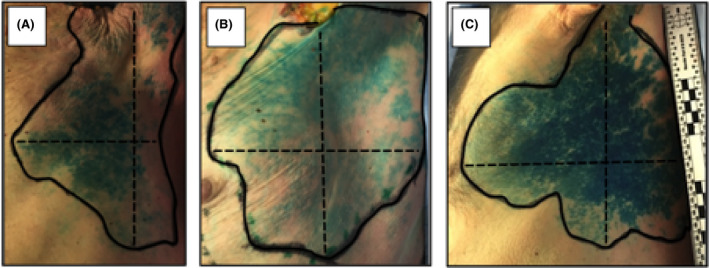
Design and size of skin islands. Three examples of a left stained skin islands are shown to illustrate diversity of islands. Each of the shown islands reached the axilla superiorly and the latissimus dorsi muscle posteriorly. Dotted lines indicate length and width of skin islands. (A, Case 3, 15 × 12.5 cm, 45.5 cm^2^; B, Case 6, 15 × 13 cm, 96.5 cm^2^; C, Case 2, 18 × 20.5 cm, 223.6 cm^2^)

Next, we wanted to define the area with highest probability for successful harvest of skin islands. Therefore, several anatomical reference lines and landmarks were used for description and characterisation of skin flaps. Mean distance between axilla and costal arch (ACA) was 30.8 ± 2.9 cm. Distance between costal arch (CA) and inferior border (IB) of stained skin island was 12.4 ± 1.8 cm (CAIB; range 9‐16 cm). On the other hand, mean distance between axilla (A) and superior border (SB) was 3.3 ± 2.7 cm (ASB; range 0‐8.5 cm). In 25% of cases (five out of 20), skin islands reached superiorly to axilla. To simplify flap harvest, we described superior (ASB) and inferior border (CAIB) of stained skin islands related to ACA, which was used as marker for the length of hemithorax. With respect to ACA, superior and inferior borders were found between the upper 0%‐29.3% of hemithorax, calculated from axilla, and the lower 32.8%‐48.3% of hemithorax, calculated from CA respectively. Thus, in craniocaudal direction, all skin islands were located within the upper 29.3%‐51.7% of hemithorax. In 90% (18 out of 20) and 30% (six out of 20) of cases, skin islands reached or even surpassed latissimus dorsi muscle (posterior axillary line) posteriorly by one to two fingerbreadths. Anteriorly the midclavicular line and nipple were reached but not passed in three cases (Table 2; Figure 3).

**Table 2:** Flap localisation according to anatomical landmarks

Case	Vertical extent					Horizontal extent	
	Total	Inferior		Superior		Posterior	Anterior
	ACA	CAIB		ASB		Latissimus dorsi muscle	Nipple
	cm	cm	%	cm	%		
1	29	13	44.8	2	6.9	+	−
2	28	12	41.9	0	0	+	+
3	32	13	40.6	4	12.5	−	−
4	29	10.5	36.2	8.5	29.3	+	−
5	30	13	43.3	6.5	21.7	+	−
6	26	11.5	44.2	0	0	+	+
7	26	9	34.6	2	7.7	+	−
8	33	16	48.5	4	12.1	+	−
9	36	15	41.7	6	16.5	+	−
10	36	13	36.1	6.5	18.0	+	−
11	32	13	40.6	2	6.3	−	−
12	32	10.5	32.8	8.5	26.6	+	−
13	29	11	37.9	0	0	+	−
14	31	11	35.5	0	0	+	+
15	32	12	37.5	0	0	+	−
16	27.5	10.5	38.2	2.5	9.1	+	−
17	34	15	44.1	3	8.8	+	−
18	33	12	36.4	6	18.2	+	−
19	33	13.5	40.9	5.5	16.6	−	−
20	29	14	48.3	1.5	5.2	+	−

The distance between axilla and costal arch (ACA), measured within anterior axillary line, has been used as surrogate marker for size of hemithorax. Distances between costal arch and inferior border (CAIB) and axilla and superior border (ASB) of stained flap islands are indicated to determine vertical extend. Distances are indicated as centimeter (cm) and percentage (%). To assess the horizontal extend of skin islands we evaluated whether anterior border of latissimus dorsi muscle (posterior) and nipple (anterior) were reached.

#### 3.1.3 Flap harvest

**Figure 4:**
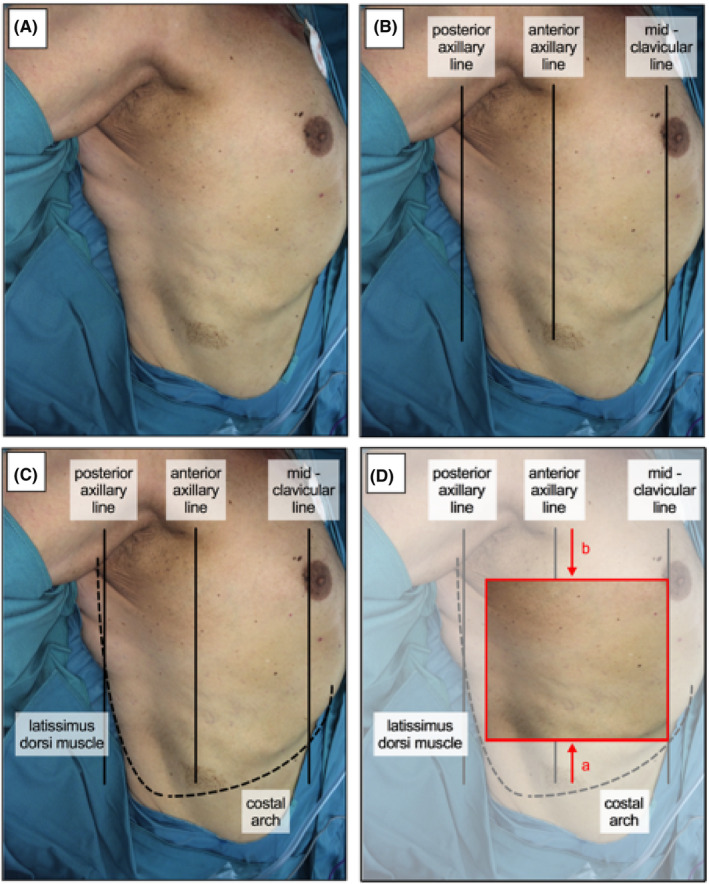
Harvest of myocutaneous serratus anterior free flap. The right hemithorax of a 65‐y‐old male patient is shown (A). Anatomical reference lines, such as posterior axillary line, anterior axillary line and midclavicular line have been indicated (B) and latissimus dorsi muscle and costal arch are shown (C). The best area (red rectangle) for flap harvest of myocutaneous serratus anterior free flap is marked (D). The midclavicular line represents the anterior and latissimus dorsi muscle the posterior border, which should not be passed. A minimal distance between costal arch and inferior border (a) and axilla and superior border (b) of 16 and 8.5 cm should be kept respectively

Accordingly, some considerations for flap design of myocutaneous SAFF are necessary. First, skin islands should not pass posterior axillary line posteriorly. Second, flap harvest should start posteriorly from latissimus dorsi muscle in anterior direction and inferior to inframammary crease, without passing midclavicular line, to gain maximal flap size (width) in anterior‐posterior direction. Within our study, width of skin islands ranged from 6 to 20.5 cm. In vertical or craniocaudal direction, a mean distance from at least 10‐12.4 ± 1.8 cm or 40.1% ± 4.4% of hemithorax should be maintained from CA. Superiorly, skin islands could reach up to axilla but a minimal distance of 3.3 ± 2.7 cm or 10.8% ± 0.9% of hemithorax, calculated from axilla, might be wise in consideration of possible wound infections at the donor side. However, highest likelihood of successful flap harvest will be found if superior and inferior border of skin islands were set at 29.3% and 51.7% of hemithorax in craniocaudal direction (calculated from axilla), and if posterior border did not pass latissimus dorsi muscle posteriorly and anterior axillary line anteriorly (Figure 4).

### 3.2 Clinical implications

#### 3.2.1 Study cohort

Five patients, comprising three males and two females, with a mean age of 59 years, received pharyngeal reconstruction after laryngopharyngectomy due to a stage IVa hypopharyngeal SCCs. Among them, two underwent salvage and three primary surgery. A Montgomery salivary bypass tube was used in all cases. Flap survival, occurrence of PCF, distal stricture and swallowing function were used as main outcome parameters (Table 3). Swallowing function was assessed by barium swallow test and additionally by fiberoptic endoscopic evaluation of swallowing (FEES).

**Table 3:** Laryngopharyngectomy and reconstruction with myocutaneous serratus anterior free flap

Nr	Sex	Age	Surgery	Size	Flap failure	PCF	Swallowing
1	F	54	Salvage laryngopharyngectomy	9 × 7	Yes	Yes	PEG
2	M	56	Laryngopharyngectomy	10 × 7	Yes	Yes	Normal
3	M	65	Salvage laryngopharyngectomy	10 × 9	No	No	Normal
4	M	61	Salvage laryngopharyngectomy	7 × 7	No	No	Normal
5	F	59	Laryngopharyngectomy	9 × 9	No	No	Normal

F, female; M, male; PEG, percutaneous endoscopic gastrostomy; SAFF, serratus anterior free flap.

Five patients underwent laryngopharyngectomy due to T4a squamous cell carcinomas of the hypopharynx. Three patients had tumour recurrence (salvage surgery) and the remaining two had primary diseases. Reconstruction of neopharynx was done with myocutaneous SAFF. Size of skin islands (cm), occurrence of flap failure or percutaneous fistula (PCF) and swallowing function are indicated.

#### 3.2.2 Flap failure

Mean area of skin islands was 9 × 7.8 cm ranging from 7 × 7 cm up to 10 × 9 cm, respectively. Among our cohort, one patient developed immediate intraoperative flap loss and another patient developed partial necrosis of the skin island within postoperative course.

In particular, immediate intraoperative flap loss occurred in a cachectic 54‐year‐old female patient with a history of follicular thyroid carcinoma and adjuvant radiotherapy who underwent salvage surgery (Case 1). A right 9 × 7 cm measuring myocutaneous SAFF was initially harvested for primary reconstruction of neopharynx. Intraoperatively, immediate vascular occlusion of the arterial anastomosis occurred several times, which forced us to remove SAFF and to harvest left anterolateral thigh (ALT) for reconstruction. Similarly to SAFF, immediate occlusion of arterial anastomosis occurred, so we finally end up with using a myocutaneous pectoralis major flap (PMF) and skin graft for closure.

Partial necrosis of myocutaneous SAFF appeared in a 56‐year‐old male (Case 2) who underwent primary laryngopharyngectomy. A right SAFF with a 10 × 7 cm measuring skin island was successfully used for closure and reconstruction of the neopharynx. Because of a postoperative abstinence delirium on day 2‐4, the patient removed several drainages and extensively manipulated on tracheal cannula and subsequently cervical anastomosis. Within endoscopic examination, we found a partial necrosis with defect of the skin island measuring 2 × 2 cm on postoperative day 7, which was most likely caused by the mechanic stress on the anastomosis. Therefore, it was most necessary to perform a reoperation to remove the necrotic area and to cover the defect with a PMF.

#### 3.2.3 Percutaneous fistula and swallowing

Those patients with flap failure also developed PCFs (two out of five). Especially the female patient developed PCF on postoperative day 14 and another fistula on postoperative day 28. It was necessary to cover both PCFs with PMFs. Conversely, no surgical intervention was necessary for the male patient and PCF closed spontaneously. After spontaneous closure of PCF, the further postoperative course of the male patient was uneventful. In strong contrast, the female patient developed tracheoesophageal fistula 6 months after surgery, which was recently closed with a regional skin flap. Moreover, due to stenosis of the neopharynx and upper part of oesophagus and the recently covered tracheoesophageal fistula, she received nutrition via percutaneous endoscopy gastrostomy (PEG) tube.

Conversely, swallowing function was inconspicuous in the remaining four patients with uneventful postoperative course without signs of strictures of neopharynx and normal oral nutrition.

## 4 DISCUSSION

The SAFF has a great versatility in reconstruction of the head and neck due to easy harvest, low donor site morbidity, constant vascular anatomy with long vascular pedicle and especially due to its pliability.^6^ The SAFF has been already used for reconstruction of skull base,^14^ scalp,^5^ oral cavity^7^ or as myocutaneous flap for facial reconstruction.^2^ Nonetheless, the myocutaneous SAFF has been rarely used for head and neck reconstruction and boundaries as well as limitations for harvest of skin islands have not been defined so far.

Although Merle et al^15^ stated that the use of SAFF as myocutaneous free flap might be impossible due to lack of skin perforators, several authors independently demonstrated direct connections between TDA and intercostal perforators to the skin.^2^, ^10^, ^13^, ^16^ Pittet et al (2006) first described boundaries for the harvest of skin islands of myocutaneous SAFF for facial reconstruction. Skin islands were limited superiorly by the inframammary crease, posteriorly by the anterior border of latissimus dorsi muscle, anteriorly approximately 2 cm medial to the midclavicular line and inferiorly by the ninth rib.^2^ Within this area, skin islands were taken randomly depending on clinical necessity with flap sizes ranging from 35 to 157.5 cm^2^, widths of 4.5 to 10.5 cm and lengths of 8 to 15 cm respectively.^2^

These recommendations are partially concordant with our results. Similarly, we set posterior and anterior boundaries at the latissimus dorsi muscle and midclavicular line respectively. Moreover, we set superior boundary 3‐5 cm (3.3 ± 2.7 cm) or 10.8% ± 0.9% of hemithorax inferior to axilla. This was in strong contrast to Pittet et al (2006) who defined superior boundary significantly lower at inframammary crease. We believe that infraaxillary area should be taken into consideration for flap design to gain additional size that might be lost if superior boundary would be set at the level of inframammary crease. Our adapted and extended boundaries might be an explanation for the significant higher lengths (10‐21 cm vs 8‐15 cm) and widths (6‐20.5 cm vs 4.5‐10.5 cm), resulting also in larger areas of skin islands (38.4‐223.6 cm^2^ vs 35‐157.5 cm^2^), in our study compared to Pittet et al (2006).

Although we found stained skin paddles in all cases, there was a great diversity of flap sizes ranging from 38.4 cm^2^ to almost six‐time larger flaps of 223.6 cm^2^. There were no statistically significant differences between female and male specimens or in left compared to right hemithoraces. Importantly, flap size did not significantly differ between paired specimens. According to our data, superior border should be set within the first to second third of hemithorax (>29.3%), while inferior border should be set within the first half of hemithorax (<51.7%), calculated from axilla, to achieve highest likelihood for successful flap harvest. Skin islands showed diverse flap designs, including almost circular or oval islands to finger‐shaped flaps.

Despite all considerations, we could demonstrate that myocutaneous SAFF represents an additional option for pharyngeal reconstruction in patients who underwent laryngopharyngectomy. Diverse fasciocutaneous flaps, including radial forearm free flap (RFFF) and anterolateral thigh (ALT) flap or visceral flaps, such as jejunum flap or gastro‐omental free flap, have been described for reconstruction of pharynx. Pharyngocutaneous salivary fistula and distal stenosis rates are reported as main outcome parameters after pharyngeal reconstruction. Mean PCF and distal stenosis rates of 15% and 9% were described for patients with ALT, which was lower compared to RFFF with 20% and 10% or gastro‐omental free flap with 16% and 22%, but similar to jejunum flap with 12% and 11% respectively.^17^ Despite similar PCF and stenosis rates between ALT and jejunum free flap, ALT is superior with regard to length of operation, donor side morbidity or tolerability of postoperative radiotherapy. The latter is significantly worse for jejunum free flaps compared to ALT flaps.^17^, ^18^ Consequently, ALT flap is primarily used for pharyngeal reconstruction followed by RFFF.^17^

Although complication rates are higher in patients who underwent salvage laryngopharyngectomy, salvage surgeries represent the only curative treatment option for patients with recurrent hypopharyngeal carcinoma. Chen et al (2013) reported on 33 patients with recurrent hypopharyngeal carcinoma who were reconstructed with ALT flap. They reported on higher rates of PCF and distal structures of 42.4% and 27.3% compared to 15% and 9% in patients who underwent primary surgeries.^17^, ^19^ Moreover, Fakhry et al (2013) reported on complications in 28% of cases (six out of 21 cases) if RFFF was used. Consequently, they recommend to additionally use pedicled PMF to reduce the risk of PCF, for protection of vessels and to foster healing of small fistulae and microleaks.^20^ Altogether, significantly higher rates of PCF are reported for RFFF compared to ALT flap (56.6% vs 30.2%) for pharyngeal reconstruction after salvage laryngopharyngectomy.^21^

So far, the SAFF has been only used as free muscle flap in few cases for reconstruction of pharyngoesophageal defects or as vascularised free muscle flap as a patch during laryngectomy.^4^, ^6^ To our knowledge, this is the first description of a series of myocutaneous SAFF for pharyngeal reconstruction after primary or salvage laryngopharyngectomy. Notably, favourable outcome has been reported with respect to PCF and distal strictures, if fasciocutaneous free flaps were used in conjunction with salivary bypass tubes.^22^ Therefore we used Montgomery salivary bypass tube in all cases. Within our cohort, mean length and width of skin islands were 9 ± 1.1 cm and 7.5 ± 1.1 cm, which were similar compared to RFFF (10.2 ± 1.8 cm and 8.5 ± 1.3 cm) and ALT flap (12.2 ± 3.8 cm and 10.6 ± 1.6 cm).^21^ Primary closure of donor side was achieved in all of our cases. This represents a significant benefit compared to RFFF, in which skin grafts are always necessary and even in 21% of myocutaneous ALT flaps, skin grafts are necessary.^21^

In our case series, three patients had uneventful postoperative courses without PCF, distal strictures, flap failure and normal oral nutrition. One undernourished female patient who underwent salvage surgery, experienced immediate intraoperative flap loss of SAFF and ALT flap and it was necessary to use pedicled myocutaneous PMF for final reconstruction. Acute malnutrition, indicated by low albumin and low BMI, represents an established poor prognostic factor for free flap survival. Low preoperative serum albumin concentrations were reported to be associated with a 4‐fold increased risk for flap failure.^23^ Whether previous radiotherapy and/or malnutrition were responsible for flap loss remains unclear. Moreover, one male patient developed partial necrosis (2 × 2 cm) of the myocutaneous SAFF with defect of neopharynx because of extensive manipulation on cervical anastomoses within postoperative delirium, which needed to be covered with PMF. The myocutaneous PMF is recommended as second choice in case of free flap failure and for therapy of PCF combined with free flaps.^17^

Final conclusions regarding use of myocutaneous SAFF for pharyngeal reconstruction after laryngopharyngectomy are significantly limited due to the small number of our case series. However, the pre‐clinical data of the anatomical part of the study, which have been transferred into clinical routine, represent the strength of the study. Nonetheless, we could show that myocutaneous SAFF represents an additional option for pharyngeal reconstruction. Of course, further studies with larger patient numbers are warranted to elucidate the reliability and versatility of myocutaneous SAFF for pharyngeal reconstruction.

## 5 CONCLUSION

In conclusion, SAFF has a reliable skin island of appropriate size that can be used for pharyngeal reconstruction after laryngopharyngectomy, when specific anatomic boundaries are considered for flap harvest.

## CONFLICT OF INTEREST

The authors have no funding, financial relationships or conflicts of interest to disclose.
